# Crystalline Nephropathy With High-Dose Methotrexate in a Patient With Primary CNS Lymphoma: A Case Report

**DOI:** 10.7759/cureus.26052

**Published:** 2022-06-17

**Authors:** Faria Latif Sami, Hasan Hammo, Ambarish Athavale

**Affiliations:** 1 Internal Medicine, John H. Stroger Jr. Hospital of Cook County, Chicago, USA

**Keywords:** acute kidney injury, glucarpidase, crystalline, nephrotoxicity, methotrexate

## Abstract

Methotrexate (MTX) is a folate antimetabolite used in the treatment of several malignancies and rheumatologic diseases. It is metabolized in the liver and excreted via the kidneys. Several adverse effects of MTX have been noted, including bone marrow suppression, mucositis, and hepatic and renal dysfunction. Close monitoring of drug levels, concurrent leucovorin administration, and urinary alkalization with aggressive hydration are some steps taken to prevent these unfavorable outcomes.

We describe a case of a patient with primary CNS lymphoma undergoing chemotherapy with high-dose methotrexate (HD-MTX) who developed methotrexate-induced crystalline nephropathy despite preventative measures. Birefringent needle-shaped crystals were demonstrated under polarized light in the urine sample in the setting of acute kidney injury (AKI). The slow decay curve of MTX causing renal and hepatic dysfunction was an indication to start glucarpidase, and a subsequent rapid decline in MTX levels with improvement in AKI was observed.

Methotrexate-induced crystalline nephropathy results from damage to the renal tubules, which in most cases is reversible. Patients with a slow decline in MTX levels may be candidates for treatment with glucarpidase, a recombinant form of carboxypeptidase G2, to allow for rapid MTX breakdown and clearance. Hemodialysis is another available treatment option for patients who develop these adverse effects.

## Introduction

As the name suggests, crystalline-induced nephropathy is acute or chronic kidney damage caused by the formation of crystals in the renal system. Several medications are known in the literature to cause this unfavorable outcome. The proposed pathophysiologic mechanisms include the supersaturation of the treatment drug in small urine volumes and/or drug insolubility at extremes of urine pH causing the medication to precipitate as crystals [1]. Formed crystals can be toxic to the renal tubules or may cause injury from direct mechanical insult. Methotrexate (MTX) is one of the drugs rarely known to cause crystalline-induced nephropathy with a handful of reported cases. It is a folic acid antagonist commonly used in the management of various autoimmune disorders and malignancies, resulting in acute kidney injury (AKI) in 2% of patients treated with high-dose methotrexate (HD-MTX) through both precipitation and direct tubular toxicity [1,2]. We present a case of a patient with primary CNS lymphoma treated with HD-MTX with a complicated course of treatment secondary to HD-MTX-induced crystalline nephropathy. Methotrexate crystals were visibly evident on urine microscopy, an occurrence of underestimated prevalence [3]. We also discuss the use of glucarpidase, a carboxypeptidase metabolizer of MTX, which has been shown to significantly reduce serum MTX levels by expediting the clearance of the medication and ultimately resulting in the resolution of AKI [4].

## Case presentation

This case is of a patient with a known medical history of hypertension, diabetes mellitus, Waldenstrom's macroglobulinemia complicated by central retinal artery occlusion, and primary CNS lymphoma diagnosed on brain biopsy.

He was started on a chemotherapy cycle after the diagnosis of primary CNS lymphoma with a chemotherapy regimen containing high-dose methotrexate (HD-MTX), rituximab 375 mg/m^2^ = 720 mg, leucovorin 100 mg/m^2^ = 200 mg (IVPB in 100 mL NS over 10 minutes every six hours until methotrexate level is less than 0.05), and temozolomide 300 mg/day.

**Figure 1:**
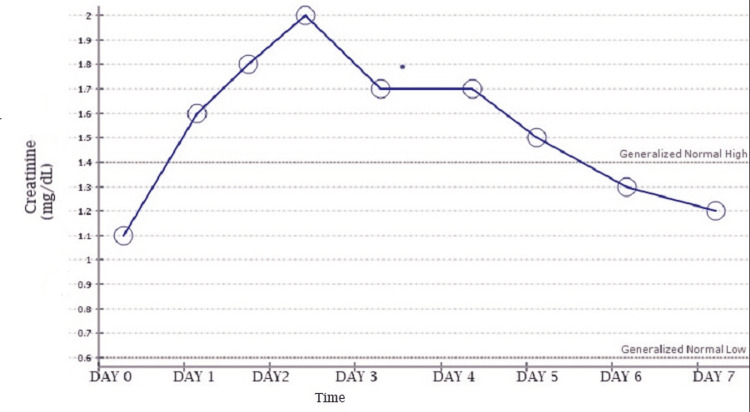
Creatinine trend

**Figure 2:**
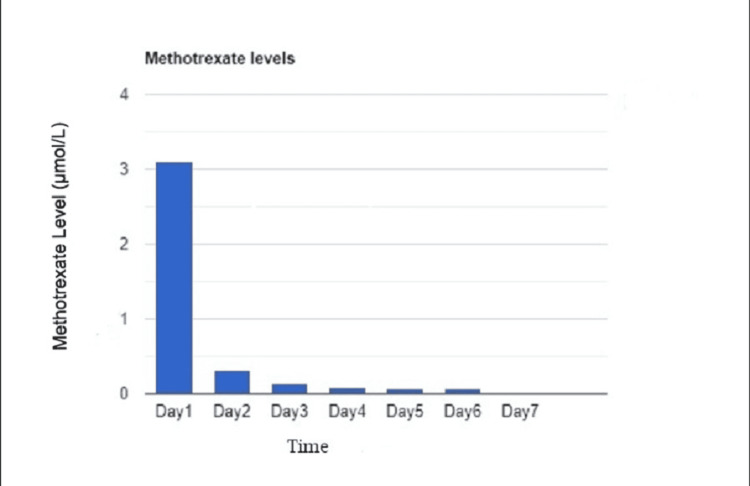
Methotrexate levels after administration on day 0

Before the initiation of chemotherapy, he was noted to have a baseline creatinine of 1.1 mg/dL. On the day after the administration of HD-MTX, creatinine elevation was noted with a peak of 2.0 mg/dL in two days. Gradual return to baseline was observed over the next five days with continued intravenous hydration and urine alkalization, as seen in Figure 1. Methotrexate levels correlated at 98 µmol/L on day 0 (day of drug administration), with a rapid downtrend as shown in Figure 2. No further renal workup was done during this admission.

The patient was admitted for the second cycle of chemotherapy 20 days later and received HD-MTX on day 0. Within 24 hours, a creatinine bump to 1.6 mg/dL was noticed, correlating with MTX levels of 120 µmol/L. Despite adequate urine alkalization with a urine pH of 8 to prevent MTX nephrotoxicity, we noticed an up-trending creatinine peaking at 4.7 mg/dL in six days, indicating an ongoing acute kidney injury (AKI). Methotrexate levels were closely monitored during this time and showed a downtrend from a peak of 120 µmol/L within 24 hours after administration to 10 µmol/L at 48 hours and 1.9 µmol/L at 72 hours. Urinalysis during this time showed a urine pH of 8, as urine alkalization and adequate hydration was achieved with 0.45% NaCl 1000 mL with 100 mEq sodium bicarbonate at 125 mL/hour. During his stay in the hospital, the patient also developed brief hypernatremia with sodium levels of 150 mEq/L and metabolic alkalosis with bicarbonate levels ranging between 30 and 41 mEq/L. Continuous intravenous fluid was deemed mildly hypernatremia and alkalotic and was eventually replaced with three ampules (50 mEq each) of NaHCO3 in 1 L of Dextrose 5% at the same rate, resulting in gradual correction of electrolytes.

**Figure 3:**
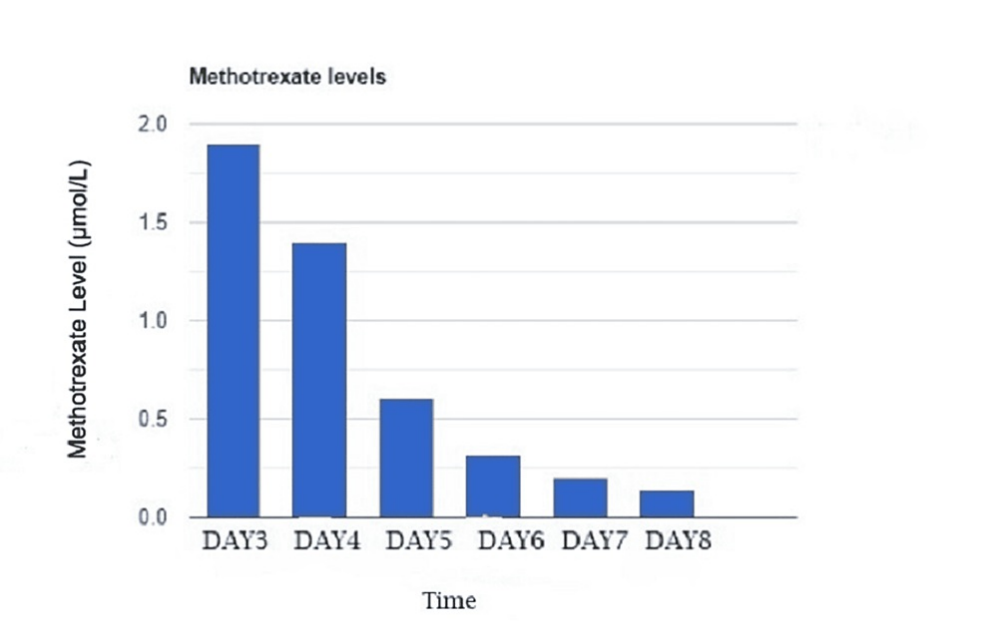
Methotrexate levels after the administration of glucarpidase on day 3

Methotrexate level at 72 hours was 1.9 µmol/L, at which point the patient was observed to reach a creatinine of 3.4 mg/dL. Liver biomarkers showed an acute elevation of AST from 28 U/L to 48 U/L, and total bilirubin rose from 0.6 mg/dL up to 0.8 mg/dL. The risk for progressive AKI and hepatic dysfunction (also likely from MTX toxicity), given the delayed decay curve of MTX, was an indication to start glucarpidase at 72 hours (day 3). He received glucarpidase 4000 units two hours after and two hours before leucovorin infusion, after which MTX levels continued to downtrend to 0.14 µmol/L on day 8 of chemotherapy as shown in Figure 3.

This patient was originally scheduled to receive leucovorin until the methotrexate level was less than 0.05 µmol/L as part of the chemotherapy regimen. However, glucarpidase is a reduced folate agent that diminishes the action of leucovorin by competing with MTX for the active site. Therefore, the administration is usually timed outside a two-hour window before and after the administration of leucovorin.

**Figure 4:**
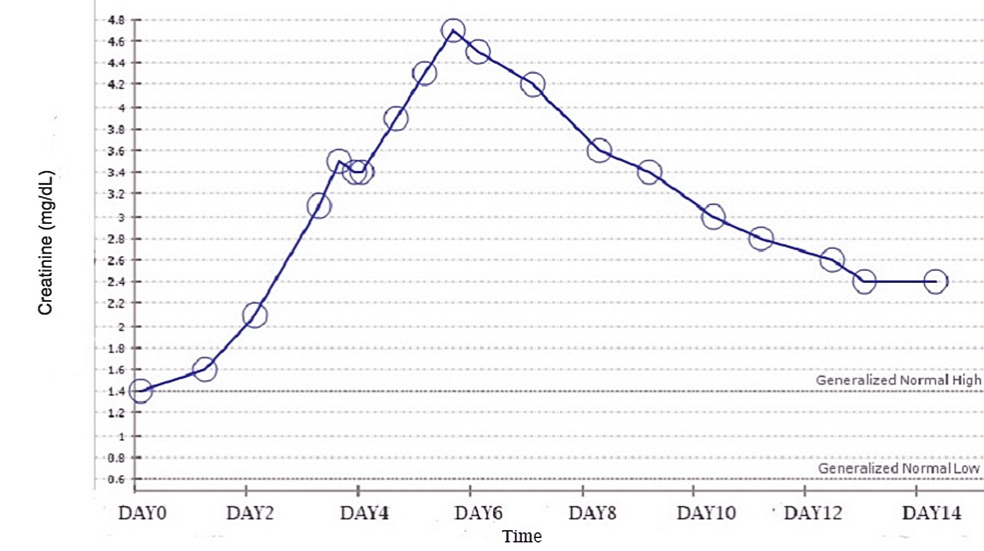
Creatinine trend with glucarpidase administration on day 3

Creatinine continued to rise to 4.7 mg/dL until day 4 of chemotherapy when the effects of glucarpidase started becoming evident with a subsequent dramatic improvement in AKI as shown by the creatinine trend in Figure 4.

The patient remained completely asymptomatic and continued to have good urine output, averaging about 1.5 L daily. On examination, the patient was observed to be euvolemic throughout his stay. No jugular venous distention and pulmonary or pedal edema findings were noted.

**Figure 5:**
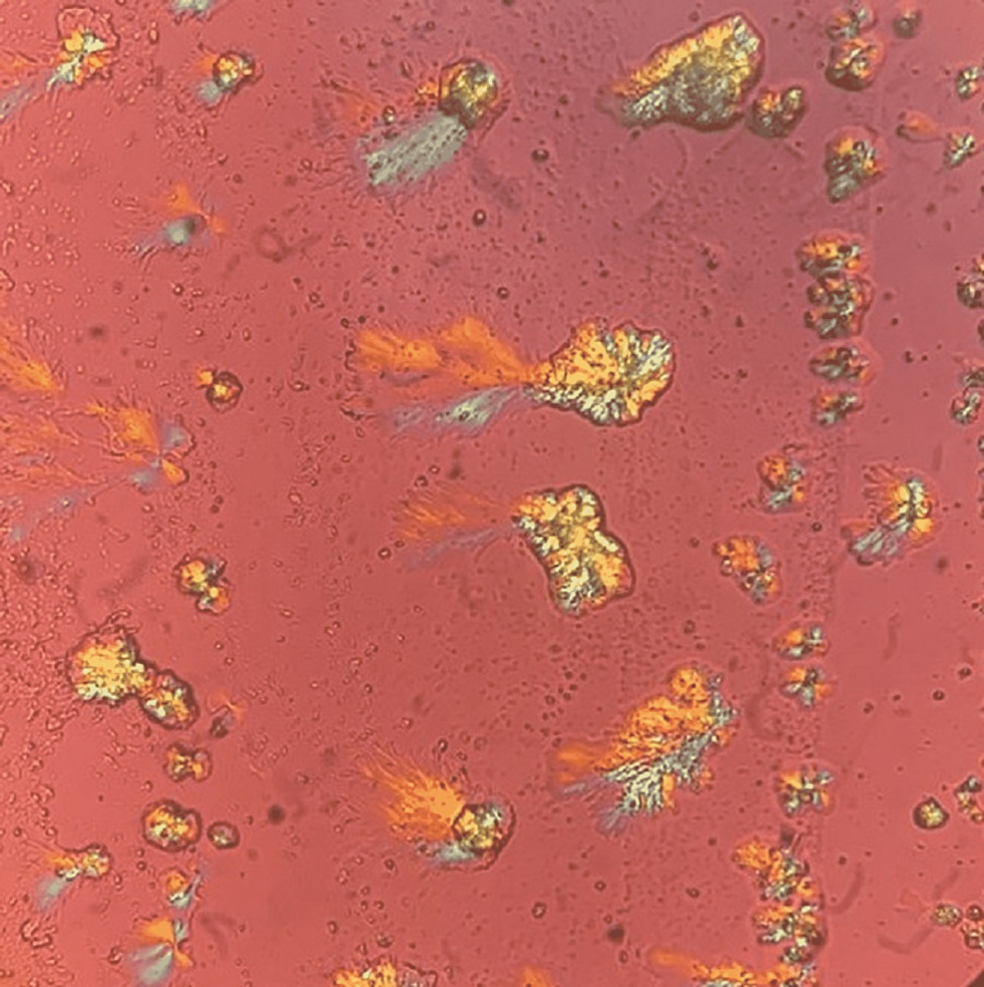
Methotrexate crystals with positive birefringence under polarized light

**Figure 6:**
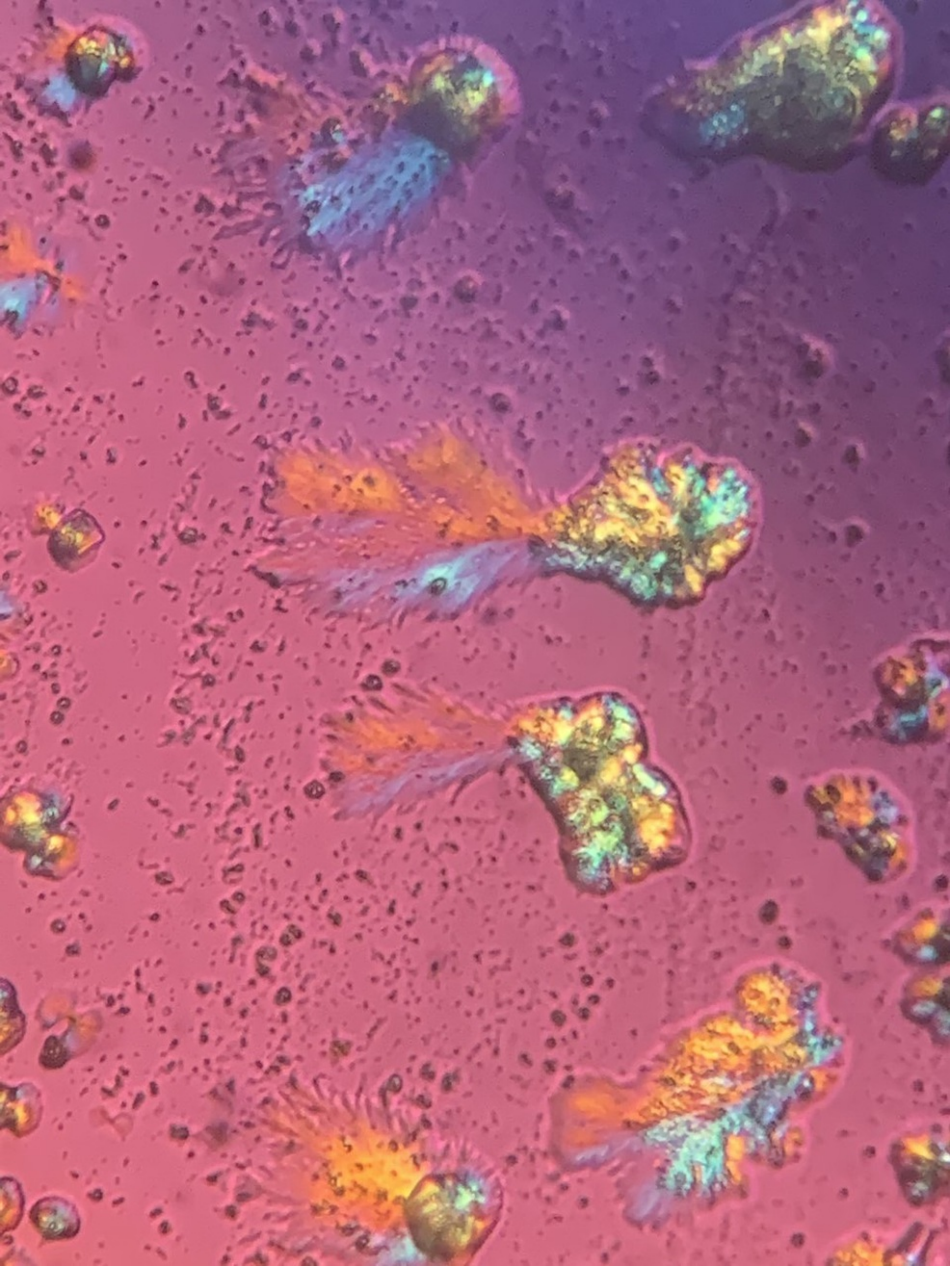
Methotrexate crystals with positive birefringence under polarized light

Urine microscopy was done as part of the workup for AKI. It was remarkable for needle-shaped, brown-colored crystals that appeared to be collected together in an annular pattern. The crystals demonstrated birefringence under polarized light as shown in Figure 5 and Figure 6.

## Discussion

Methotrexate (MTX), an antifolate metabolite, is a useful anticancer medication for the treatment of hematological malignancies. As it is metabolized in the liver and cleared by the kidneys, these organs are at risk of injury when exposed to high doses, resulting in renal and hepatic dysfunction. Apart from this, MTX is also known to cause myelosuppression, mucositis, immune function decline, pulmonary fibrosis, and fetal abnormalities. The risk of these side effects dramatically increases at higher doses such as those used as part of chemotherapy regimens, and about 2%-12% of these patients are likely to develop acute kidney injury (AKI) [5].

MTX crystal formation in renal tubules, drug-induced afferent arteriolar constriction reducing the glomerular filtration rate, and direct tubular toxicity are all known mechanisms by which MTX can cause AKI [2]. However, MTX crystalline nephrotoxicity is a reversible form of AKI, where peak creatinine elevations are most frequently observed during the first 5-6 days of treatment initiation, and subsequent return to baseline creatinine can be expected in up to four weeks [6]. These patients may remain asymptomatic or may develop decreased urinary output at the onset of AKI. Patients may also be observed to have a slow decline in MTX levels due to AKI, mitigating the renal clearance of the drug. Patients with AKI may need up to eight days of median time for MTX clearance, whereas those with normal renal function only need five days on average [7].

Some established risk factors of developing acute kidney injury when started on HD-MTX are underlying renal dysfunction, volume depletion, urine acidity (pH <8), and various other drug interactions [5]. It has been proposed that low albumin levels (under 3.6 g/dL) and creatinine above 0.9 mg/dL at baseline also increase the likelihood of the adverse effects of MTX [7].

MTX should be suspected as the possible culprit for AKI in the setting of elevated creatinine with concurrent elevated methotrexate levels and confirmed with visualization of methotrexate crystals under light microscopy of urine or on kidney biopsy revealing intratubular methotrexate crystals [8].

Adequate hydration and urinary alkalization before drug administration are supportive measures to prevent nephrotoxicity in patients at high risk of renal injury and receiving a high dose of methotrexate. During chemotherapy, leucovorin is also administered in conjunction with HD-MTX with continuous monitoring of both MTX levels and renal function in serum [5]. Leucovorin rescue is thought to work around the principle of continued DNA synthesis in noncancerous cells by supplying reduced folate to bypass the MTX block in these cells.

In patients who develop methotrexate-induced nephrotoxicity and delayed methotrexate clearance, acetazolamide can also be used to achieve a urine pH >7, and the administration of high-dose leucovorin helps mitigate the ongoing damage [5].

Other modalities that can be used to alleviate the risk of worsening renal function and protect against other side effects include glucarpidase and hemodialysis to remove MTX [5,9,10].

Glucarpidase, a recombinant bacterial carboxypeptidase that cleaves MTX, is highly effective in preventing life-threatening methotrexate toxicity in patients with AKI. One dose of 50 U IV over five minutes has been shown to lower plasma MTX concentrations within 15 minutes of administration by >97%. It is however ineffective against intracellular stores and thus must be followed by leucovorin for continuous and effective MTX depletion [5,11].

Most acute kidney injuries are reversible with these various lines of treatment; however, they are associated with an increased mortality rate [7]. For this reason, it becomes excruciatingly important to explore the prevalence and risk factors for MTX-induced nephrotoxicity and the effectiveness of available treatment modalities.

## Conclusions

Methotrexate-induced crystalline nephropathy is a reversible but under-recognized adverse effect of MTX. It is characterized by acute tubular injury after the administration of high-dose methotrexate secondary to MTX crystal formation in the renal tubular system. Scattered intratubular needle-shaped, golden-brown crystals arranged in annular structures, birefringent under polarized light, can be demonstrated with urine light microscopy. MTX crystals and resulting AKI can be observed even in the absence of toxic MTX levels. Preventative measures include fluid hydration, urinary alkalization with a goal pH of greater than or equal to 8, and concurrent leucovorin administration. If acute kidney injury develops and persists despite these measures, glucarpidase can be effective in expediting the MTX clearance, although some patients may eventually become candidates for hemodialysis to avoid progressive detrimental outcomes.
